# Diagnosing and Staging of Cystic Echinococcosis: How Do CT and MRI Perform in Comparison to Ultrasound?

**DOI:** 10.1371/journal.pntd.0001880

**Published:** 2012-10-25

**Authors:** Marija Stojkovic, Kerstin Rosenberger, Hans-Ullrich Kauczor, Thomas Junghanss, Waldemar Hosch

**Affiliations:** 1 Section Clinical Tropical Medicine, Department of Infectious Diseases, Heidelberg University Hospital, Heidelberg, Germany; 2 Department of Diagnostic and Interventional Radiology, Heidelberg University Hospital, Heidelberg, Germany; Universidad Peruana Cayetano Heredia, Peru

## Abstract

**Background:**

Imaging plays the key role in diagnosing and staging of CE. The description of CE-specific imaging features and the WHO CE cyst classification is based on ultrasound. The reproducibility of the ultrasound-defined features of CE cysts is variable in MR- and CT-imaging. This is of particular importance for cysts that are not accessible by US and because of the increasing availability and overuse of CT and MR imaging.

**Methodology/Principal Findings:**

Retrospective analysis of patients with abdominal CE cysts of an interdisciplinary CE clinic who had CT and/or MRI scans performed additionally to US imaging. All images were read and interpreted by the same senior radiologist experienced in the diagnosis of CE. US, CT and MR images were staged according to the WHO classification criteria. The agreement beyond chance was quantified by kappa coefficients (κ). 107 patients with 187 CE cysts met the inclusion criteria. All cysts were assessed by US, 138 by CT, and 125 by MRI. The level of agreement beyond chance of the individual CE stages 1–4 was clearly lower for CT, with κ ranging from 0.62 to 0.72, compared to MRI with values of κ between 0.83 and 1.0. For CE5 cysts CT (κ = 0.95) performed better than MRI (κ = 0.65).

**Conclusions:**

Ultrasound remains the corner stone of diagnosis, staging and follow up of CE cysts. MRI reproduces the ultrasound-defined features of CE better than CT. If US cannot be performed due to cyst location or patient-specific reasons MRI with heavily T2-weighted series is preferable to CT.

## Author Summary

Cystic echinococcosis (CE) is a neglected parasitic disease of global distribution. The highest prevalence rates are recorded in South America, Northern and Eastern Africa, Eurasia and Australia. In non-endemic and largely high income countries CE is a disease of immigrants. Imaging plays the key role in diagnosing and staging of CE. Ultrasound (US) remains the cornerstone of diagnosis, staging and follow up of CE cysts. Translation of the ultrasound-based classification of CE into magnetic resonance imaging (MRI) and computed tomography (CT)-imaging deserves attention since there are pitfalls of great significance for the diagnosis and management of patients with CE. With an increasing use of MRI and CT-imaging also in middle income countries of which many are endemic for CE the advantages and disadvantages of these imaging modalities deserve greater attention. We present a data set of US-, MRI- and CT-investigations of patients with CE where we can demonstrate that compared to US MRI is superior to CT in diagnosing and staging CE cysts. MRI reproduces the ultrasound-defined features of CE better than CT. If US cannot be performed due to cyst location or patient-specific reasons MRI is preferable to CT.

## Introduction

Cystic echinococcosis (CE) is a neglected parasitic disease of global distribution [1], [2]. The highest prevalence rates are recorded in South America, Northern and Eastern Africa, Eurasia and Australia. In non-endemic and largely high income countries CE is a disease of immigrants.

Imaging plays the key role in diagnosing and staging of CE, whereas serology has only a minor, confirmatory role due to high rates of false negative results [3]. This is particularly the case in the early cyst stages when hydatid fluid is still tightly contained within the endocysts (cyst stage CE1) and in the final stage of involution (CE5) when cyst content is solid and the cyst wall largely calcified.

The description of CE-specific imaging features and CE cyst classification is based on ultrasound (US). A set of ultrasonographic features has been agreed upon as the imaging reference standard for diagnosing and staging CE, resulting in a standardized WHO classification based on the Gharbi US classification of 1981 [4]–[7], [17] (Figure 4).

Through ultrasound substantial progress has been made in recent years in understanding the natural and treatment driven involution of CE cysts . This to an extent that treatment decision can increasingly be based on cyst stages. By and large CE1, CE2 are regarded as “active”, CE3 as “transitional” and CE4 and CE5 as “inactive” cyst stages [6]–[9], [17]. In uncomplicated cysts, the four available treatment modalities can be assigned to these cyst stages: small early cysts, in particular CE1, can be given a trial with albendazole [10] and larger CE1 liver cysts up to a diameter of 10 cm are ideal for PAIR. For advanced cysts (CE4 and CE5) good evidence has accumulated that they can confidently be left alone (watch & wait). More critical are CE2 and the transitional cyst stage CE3b which often need surgery for definite cure. Identification of cysto-biliary and cysto-bronchial communications is critical, in particular when protoscolicidal substances are used to sterilize the endocyst and cyst content. Imaging, specifically magnetic resonance imaging (MRI) with heavily T2-weighted series [11], plays a role here, but can not completely rule out communications pre-interventionally.

Computed tomography (CT) and MR imaging features of CE cysts have also been well characterized [12]–[14]. But there is a lack of systemic comparisons between different imaging modalities in CE. We systematically searched PubMed from its beginnings until July 29^th^, 2012 with the search terms [(cystic echinococcosis or hydatid disease) and (CT or MRI or MR)] and retrieved 1351 results. Only 2 publications compared at least two of the imaging modalities US, MRI and CT. Suwan [15] compared US (N = 62) with CT (N = 25) and Taourel et al [16] CT and MRI (N = 27). Suwan found that sonography was superior to CT in the characterization of cyst content but CT was superior to sonography in detecting gas within the cysts and minute calcifications. Taourel et al concluded that MRI was superior to CT in identifying complications but did not help to characterize solid or pseudotumoral forms of CE.

The role of CT and MRI in staging CE has never been evaluated, however. This is of particular importance for cyst which are nor accessible by US and because of the increasing availability and overuse of CT and MR imaging to avoid misclassification.

We present a data set of US-, MRI- and CT-investigations of patients with CE to determine the performance of CT and MRI in comparison to the gold standard US.

## Methods

The Section of Clinical Tropical Medicine at Heidelberg University Hospital runs an interdisciplinary clinic for patients with CE since 1999. Clinical, serological and radiological data of patients attending the clinic are systematically registered and patients are followed-up for 5–10 years after completion of treatment. All images are stored in a Picture Archiving and Communication System (PACS; Centricity, Version 2.0, GE Medical Systems Integrated Imaging Solutions, Mt. Prospect, USA) since February 2000. Over the years diagnosis and treatment of patients has been highly standardized. Patients are triaged into four treatment groups: albendazole, PAIR (*p*uncture, *a*spiration, *i*njection of a scolecidal agent and *r*easpiration), surgery and watch & wait as previously reviewed [7], [8], [17].

### Patients

The patients with abdominal or soft tissue echinococcal cysts who had CT and/or MRI scans performed additionally to US imaging within a time period of three months were selected from our Picture Archiving and Communication System. Albendazole treatment before and during the 3-months period of assessment was not taken into account. In total 107 patients with 187 abdominal cysts were included into our study.

The Institutional Review Board of the University Hospital of Heidelberg has approved this retrospective analysis of clinical data and radiological examinations (reference number: 243/2011).

### Ultrasound examination (US)

All cysts (n = 187) were examined by US with conventional B mode US using a Sonoline Elegra platform (Siemens Ultrasound Division, Issaquah, Washington, USA) or a Logiq 9 platform (GE, Milwaukee, USA) with a 3.5 MHz and 7 MHz multifrequency transducer.

### Computed tomography (CT)

A total number of 138 cysts were examined by unenhanced and contrast-enhanced CT, of which 112 cysts were recorded on CTs from external radiological institutions. 26 cysts were examined in-house using different scanner generations with 4, 16, 64, and 256 slice technology (Siemens Somatom, Sensation, Definition, Forchheim, Germany; Philips Brilliance 64 and iCT, Philips Healthcare, The Netherlands). CTs of all patients were run with a routine abdominal protocol with unenhanced and contrast enhanced scans. Images were reconstructed with a slice thickness between 3 and 5 mm and overlapping reconstruction increment.

### Magnetic resonance imaging (MRI)

In total, 125 cysts were examined by MRI of which 45 cysts were digitally recorded from external radiological institutions. 80 cysts were examined in-house using 1.5 Tesla systems Magnetom Symphony equipped with a high-performance gradient system (maximum gradient strength: 30 mT/m, slew rate: 125 T/m/s) and since May 2004 on a Siemens Avanto Symphony equipped with a 40 mT/m gradient system and a slew rate of 170 T/(m/s).

The in-house MRI protocol with detailed sequence parameters is listed in Table 1.

**Table 1:** In-house MRI protocols with detailed sequence parameters.

		Symphony	Avanto
**TrueFisp** 1	TR^2^/TE^3^ [ms]	4.3/2.15	5.18/2.59
	flip angle [°]	51	80
	slice thickness [mm]	6	6
	matrix size	256×256	384×512
**T1w FLASH** 4	TR/TE [ms]	128/4.76	168/4.76
	flip angle [°]	70	70
	slice thickness [mm]	6	6
	matrix size	256×267	256×256
**T2w TSE** 5	TR/TE [ms]	3220/109	4050/112
	flip angle [°]	150	150
	slice thickness [mm]	6	6
	matrix size	256×256	256×256
**HASTE** 6	TR/TE [ms]	1400/105	1530/119
	flip angle [°]	125	160
	slice thickness [mm]	6	6
	matrix size	256×512	256×512
**post-contrast**			
**T1w 3D-FLASH**	TR/TE [ms]	3.73/1.44	3.5/1.31
	flip angle [°]	25	12
	slice thickness [mm]	2.5	3
	matrix size	343×512	384×512
**T1w 2D-FLASH**	TR/TE [ms]	157/6	142/6
	flip angle [°]	70	70
	slice thickness [mm]	8	8
	matrix size	256×256	256×256

1 TrueFisp: True Fast Imaging With Steady Precession,

2 TR: Repetition Time,

3 TE: Time to Echo.

4 FLASH: Fast Low Angle Shot,

5 TSE: Turbo-Spin-Echo,

6 HASTE: Half fourier-Acquired Single shot Turbo spin Echo, w: weighted.

### Evaluation of US, MRI and CT images

All images were interpreted by the same Board-examined senior staff radiologist (WH) experienced in the diagnosis of CE and member of WHO Informal Working Group on Echinococcosis (WHO-IWGE). US, CT and MR images were assessed using a Picture Archiving and Communication System (PACS; Centricity, Version 2.0, GE Medical Systems Integrated Imaging Solutions, Mt. Prospect, USA). CT and MR images of external radiological institutions have been imported into the PACS system. The CT, MRI and US series were separately read in random order. In the final step, the results of the three imaging modalities were matched to the patient.

The optimal window setting for analyzing the images of each case in the PACS was adjusted individually as needed. To evaluate which standard MRI sequence is best for the classification of echinococcal cysts abdominal T2w-standard sequences TrueFisp (True Fast Imaging With Steady Precession), HASTE (Half fourier-Acquired Single shot Turbo spin Echo), T2w-TSE (T2-weighted Turbo-Spin-Echo), as well as contrast enhanced T1w FLASH (Fast Low Angle Shot) or the corresponding sequences of other manufacturers than Siemens (n = 13) were evaluated in a separate session. Independent of the imaging modality cysts were staged according to WHO classification [4], [17], [18] (Figure 4).

The following thresholds for CT assessment were used: 0 to 20 Hounsfield Units (HU) to identify liquid cyst content, 20 to 130 HU for mucinous or solid content, and 130 HU or higher on unenhanced images to identify calcification. For MRI assessment liquid cyst content has been identified on T2w sequences by comparing its signal intensity with liquid content of the gallbladder or CSF in the spinal canal for reference.

### Statistical analysis

We analysed agreement between the cyst stages as determined by CT or MRI and US which was defined as the standard of reference. The agreement beyond chance was quantified by kappa coefficients. Kappa values from 0.81–1.0 were considered very good, values from 0.61–0.80 good and 0.41–0.60 as moderate [19].

Confidence intervals for the kappa coefficients were calculated using the Stata command “kapci”. As described by Reichenheim [23], the calculation is based on an analytical method in the case of dichotomous variables [20] and a bias corrected bootstrap method in the case of nominal variables [21], [22] (see Stata documentation on the command “kapci”). The number of bootstrap replications was set to 1000. A chi-square test for heterogeneity between kappa coefficients of in-domo and ex-domo MRI scans was performed in a meta-analytic framework using the Stata command “metan” [24]. The kappa coefficients and the corresponding confidence intervals estimated as indicated above were used as input for the meta-analysis.

All calculations were done in Stata versions 9.2 and 12.1 (STATA Corporation, College Station, Texas).

## Results

107 patients with 187 CE cysts met the inclusion criteria. 47 patients were female, 60 male. The age of the patients ranged from 7–78 years. The cyst localisation was: 171 liver, 6 spleen, 3 kidney, 5 peritoneum, 2 in the soft tissue. Median maximal cyst diameter was 5.5 cm ranging from 1 to 23 cm. All 187 cysts were assessed by US, 138 by CT, and 125 by MRI.

**Figure 1:**
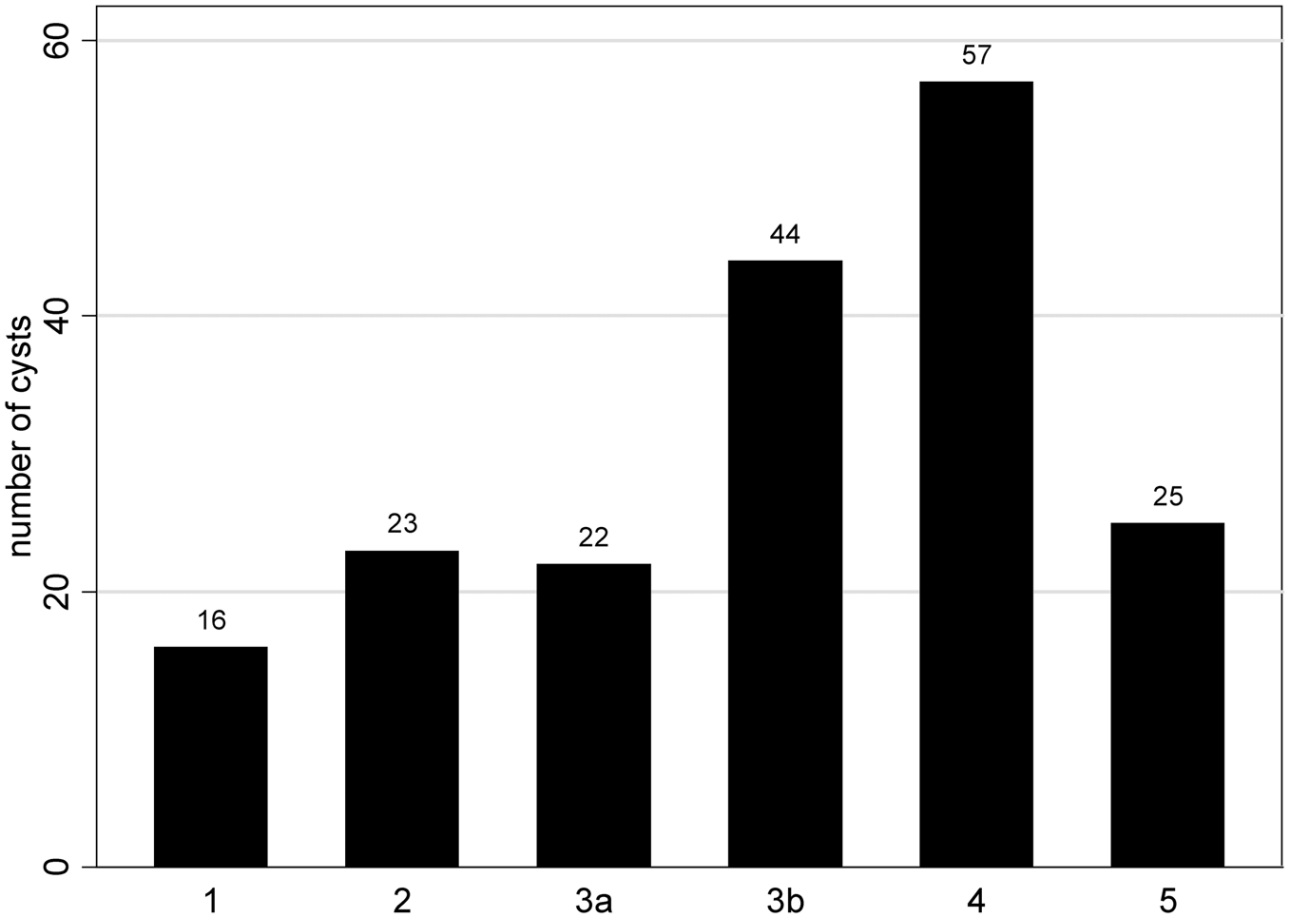
Number of cysts per WHO cyst stage (CE 1–5) as determined by US (N = 187).

Figure 1 shows the distribution of WHO cyst stages as determined by US.

**Figure 2:**
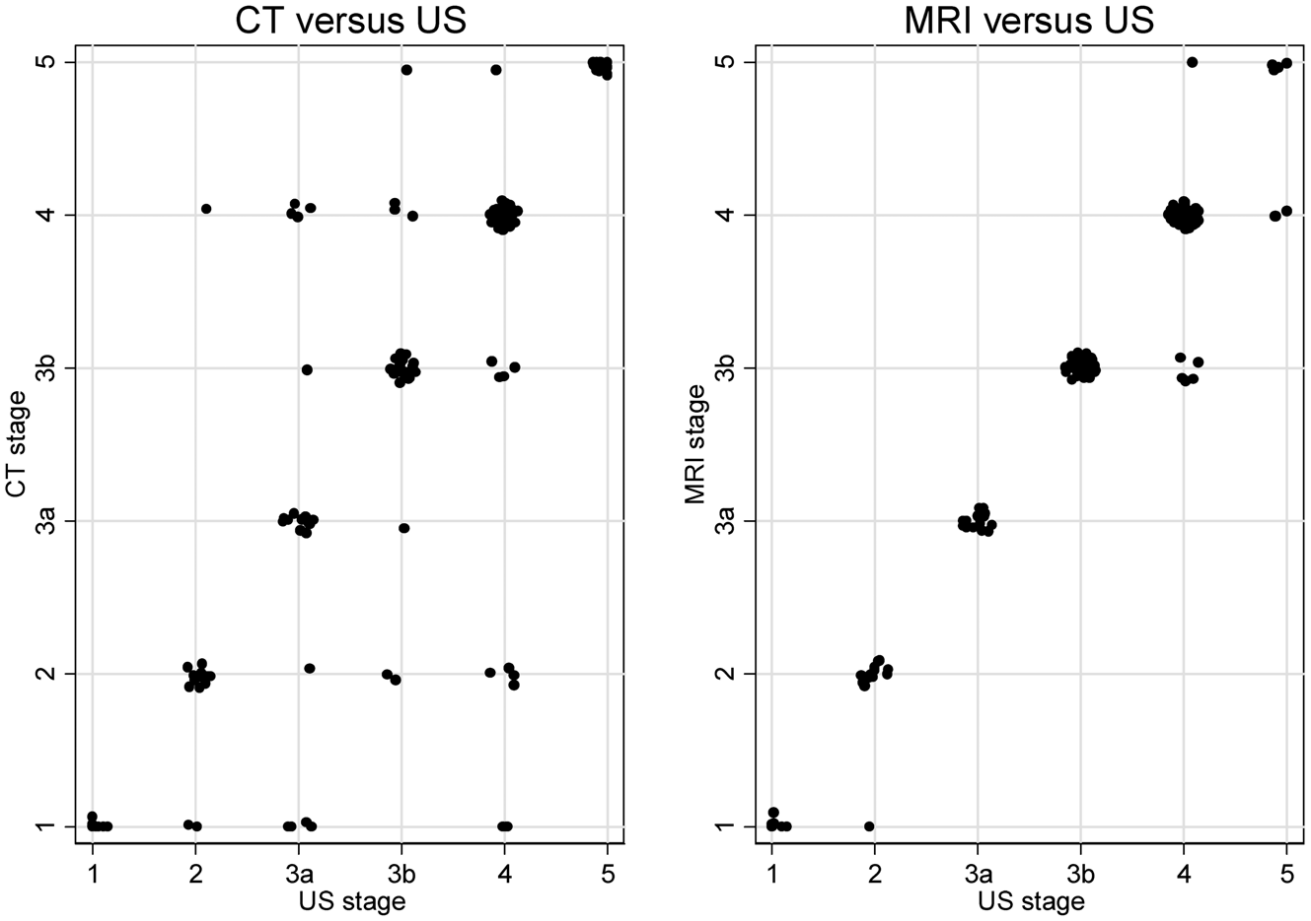
Scatter plots of the agreement beyond chance of US vs. CT and US vs. MRI.

Figure 2 shows a scatter plot of the WHO classification-based cyst staging with a level of agreement beyond chance of the individual CE stages 1–4 clearly lower for CT, with κ ranging from 0.62 to 0.72, compared to MRI with values of κ between 0.83 and 1.0. For CE5 cysts CT (κ = 0.95) performed better than MRI (κ = 0.65) (Table 2, 3).

**Table 2:** Levels of agreement and kappa coefficients for US vs. CT, US vs. MRI and US vs. different MRI sequences.

Method	N° of cysts	Agreement (%)	Expected Agreement (%)	Kappa	Std. Err.	95% CI
**CT**	138	76.09	18.40	0.71	0.0391	(0.61–0.79)
**MRI**	125	92.00	22.97	0.90	0.0460	(0.83–0.95)
**T1w-FLASH** 1	125	72.80	22.98	0.65	0.0455	(0.55–0.74)
**T2w-TSE** 2	118	81.36	21.90	0.76	0.0461	(0.68–0.85)
**TrueFisp** 3	100	86.00	24.57	0.81	0.0528	(0.72–0.90)
**HASTE** 4	120	91.67	23.06	0.89	0.0471	(0.83–0.95)

1 FLASH: Fast Low Angle Shot,

2 TSE: Turbo-Spin-Echo,

3 TrueFisp: True Fast Imaging With Steady Precession,

4 HASTE: Half fourier-Acquired Single shot Turbo spin Echo (or corresponding sequences of other manufacturers than Siemens). Differences in number of cysts are due to varieties in MRI-protocols, especially of ex-domo-patients.

**Table 3:** Levels of agreement and kappa coefficients for US vs. CT and for US vs. MRI stratified by WHO stages (defined by US).

US versus CT (N = 138)					
Cyst stage	Agreement (%)	Expected Agreement (%)	Kappa	Std. Err.	95% CI
1	92.75	80.36	0.63	0.0791	(0.43–0.84)
2	92.75	77.27	0.68	0.0844	(0.50–0.87)
3a	92.03	78.73	0.63	0.0810	(0.43–0.82)
3b	91.30	68.52	0.72	0.0850	(0.58–0.87)
4	84.78	59.70	0.62	0.0848	(0.48–0.77)
5	98.55	72.21	0.95	0.0850	(0.88–1.00)

US versus MRI (overall) (N = 125)					
Cyst stage	Agreement (%)	Expected Agreement (%)	Kappa	Std. Err.	95% CI
1	99.20	83.29	0.95	0.0893	(0.86–1.00)
2	99.20	79.49	0.96	0.0894	(0.89–1.00)
3a	100.00	77.68	1.00	0.0894	(1.00–1.00)
3b	96.00	57.29	0.91	0.0890	(0.83–0.99)
4	92.80	57.34	0.83	0.0893	(0.73–0.94)
5	96.80	90.85	0.65	0.0881	(0.33–0.97)

Cyst-stage specific kappa values: CT are more to the lower end of the category “good” (0.61–0.80), MRI at the upper end of the category “very good” (0.81–1.0).

**Figure 3:**
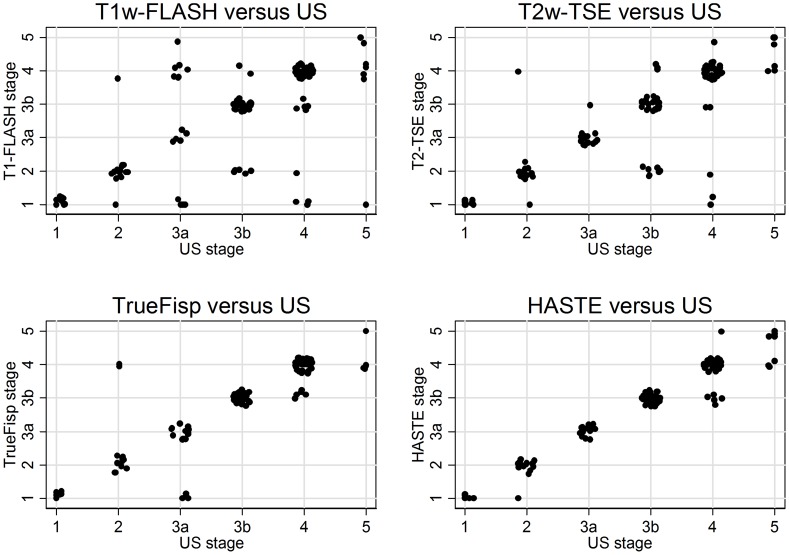
Scatter plots of the agreement beyond chance of US versus MRI. Scatter plots of the agreement beyond chance of US versus contrast enhanced T1w-FLASH, TrueFisp, HASTE and T2w-TSE MRI modes.

Comparison between US and individual MRI sequences for MRI examinations are shown in Figure 3. The highest level of agreement was found between US and HASTE with a kappa coefficient of 0.89 and US and TrueFisp with a kappa coefficient of 0.81. See Table 2 for details. The results of in-domo MRI scans run with standardized protocols and ex-domo MRI scans with varying protocols and sequence parameters have been pooled, because no significant differences were found in the level of agreement beyond chance between MRI and US (Chi2-Test for heterogeneity between kappa values of in-domo and ex-domo MRI: p = 0.395; data not shown). Differences between in-domo and ex-domo in CT examinations do not need to be considered, because of comparable imaging and reconstruction protocols.

## Discussion

Imaging plays the key role in diagnosing and staging CE cysts. The description of CE-specific imaging features and the WHO-IWGE CE cyst classification is based on ultrasound. The reproducibility of the ultrasound-defined features of CE cysts is variable in MR- and CT-imaging.

Since treatment decisions are driven by imaging it is important to know how the ultrasound-based classification of CE cysts translates into MR- and CT-imaging for cases where MRI and CT substitute for US. This is in cysts which are not accessible for US, but plays also an increasing role due to widespread availability and overuse of CT and MR imaging.

**Figure 4:**
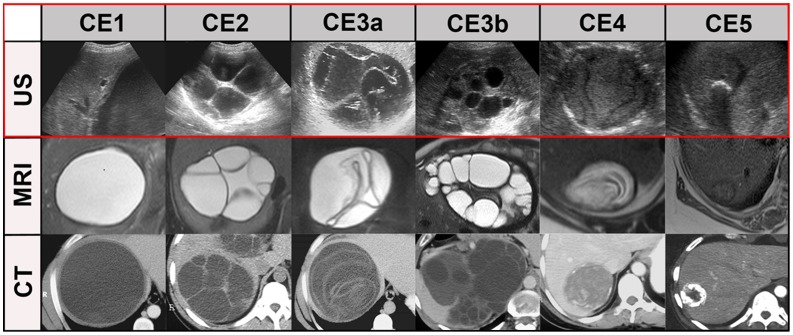
“Best case” of CT/MR imaging. CE1: unilocular, simple cysts with liquid content and often with the CE1-specific “double line sign”, CE2: multivesicular, multiseptated cysts, CE3a: cysts with liquid content and the CE3a-specific detached endocyst, CE3b: unilocular cysts with daughter cysts inside a mucinous or solid cyst matrix, CE4: heterogenous solid cysts with degenerative, CE4-specific canalicular structure of the cyst content, and CE5: cysts with degenerative content and heavily calcified wall.

**Figure 5:**
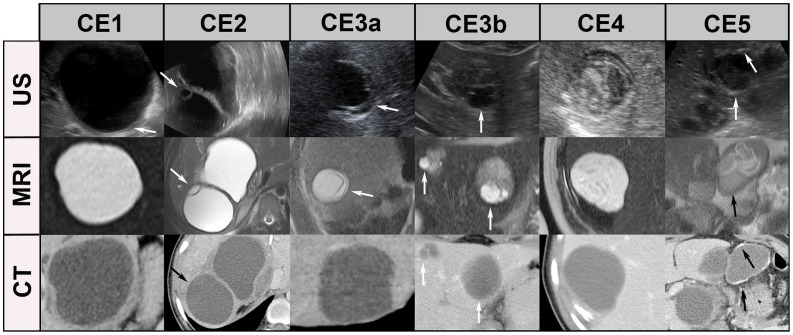
“Worst case” of CT/MR imaging. The “double line sign”, typical for CE1 is often seen in US (CE1/US, arrow), less reliably in MRI and CT. Daughter cysts and detached endocyst (“water-lily-sign”) is often missed by CTs, but clearly visible in US and MRI (see CE2, CE3a, arrows). Daughter cysts inside a solid cyst matrix are often not recognized by CT (CE3b, arrows). The CE4-specific canalicular structure is often not visible on CT images. These cysts may be misinterpreted as type CE1 cysts, i.e. staged “active” instead of “inactive”. The identification of calcifications is the domain of CT imaging. MRI does not differentiate well between thick hyaline walls and calcifications. US picks up calcifications only when a dorsal echo shadow is produced (see CE5, arrows). MRI: HASTE sequence, CT: post contrast enhanced images.

Compared to the reference standard ultrasound, the performance of MRI may be a problem in WHO cyst stages CE4 and 5, and is definitely a problem in CT imaging in a much wider range of cysts (CE1, CE2, CE 3a, b, CE4). Figure 4 and 5 show typical US, MR- and CT-images with the “best case“ for CT/MR imaging and the “worst case” for CT/MR imaging, whereby the “best case” of cyst stages CE2, CE3a,b and CE4 is rarely achieved by CT scanning.

Our data shows that cyst stages CE1 to CE4 determined by MRI compared to the reference standard US have a very good level of agreement with a kappa value between 0.83–1.00. Analysis of individual MRI sequences exhibit similar accordance. In HASTE sequences the kappa value is 0.89. T2-weighted sequences, in particular TrueFisp and HASTE sequences, detect liquid content in the cyst matrix best, i.e. daughter cysts (CE2, CE3b) and septae (CE2). In contrast cysts staged CE1 to CE4 evaluated by CT show clearly lower kappa values which range between 0.62 and 0.72.

Compared to ultrasound CT performs satisfactorily in CE5 cysts (κ = 0.95). This is due to the amount of calcifications for what CT is the diagnostic standard. In all other cysts in which the texture of the cyst matrix is of importance for classification CT performs moderately.

MRI has shortcomings in identifying details of the cyst wall, in particular calcifications which play some role in defining cyst stage CE5 with a kappa of 0.65. Cyst wall calcification is, however, not a cyst stage defining feature in itself as has been recently shown in a large data set [11]. The highly specific cyst stage defining features are features of the cyst matrix with the exception of the “double line sign” of CE1 (see Figure 4, 5) where a cyst wall feature is diagnostic. MRI and in particular heavily T2w sequences (e.g. HASTE sequences) show a performance which is comparable to US.

The superiority of MRI in comparison to CT imaging in staging CE cysts is in line with the well known observation in both the detection and characterization of focal liver lesions in general despite the fact that CT imaging provides very high spatial as well as temporal resolution, due to its superior soft tissue contrast [25], [26].

A possible limitation of our study is that there was some variation in CT and MR imaging protocols between patients due to the retrospective study design. However, when comparing the two main subpopulations, the in-house MRI scans run with standardized protocols and the ex-domo MRI scans with varying protocols and sequence parameters, no significant differences were found in the level of agreement beyond chance between MRI and US. Another limitation is that only one radiologist read the images and interobserver variation could not be assessed.

In conclusion, ultrasound remains the corner stone of diagnosis, staging and follow up of CE cysts. MRI reproduces the ultrasound-defined features of CE better than CT. If US can not be performed due to cyst location or patient-specific reasons MRI with heavily T2-weighted series is preferable to CT.

## Supporting Information

Checklist S1 (DOC)

Flowchart S1 (DOCX)
