# Supplementary material for: Diagnosing and Staging of Cystic Echinococcosis: How Do CT and MRI Perform in Comparison to Ultrasound?
Source: PLoS Negl Trop Dis. 2012 Oct 25;6(10):e1880. doi: 10.1371/journal.pntd.0001880 (PMC3493391; doi:10.1371/journal.pntd.0001880)
Supplement: Flowchart S1 — (DOCX) [file pntd.0001880.s002.docx]

36 patients have been excluded due to extraabdominal location of CE cysts and a time period of US imaging and CT and/or MRI scans of >3 months

All 138 cysts were correlated with **ultrasound**, defined as the **standard of reference**

All 125 cysts were correlated with **ultrasound**, defined as the **standard of reference**

138 cysts examined by **CT**

125 cysts examined by **MRI**

187 CE cysts
in 107 patients

143 patients with CE between 2000 and 2010
